# Short communication and clear content of geriatric recommendations matter! Evaluation of geriatric recommendation acceptance in patients aged ≥ 85 years hospitalized for cardiac conditions

**DOI:** 10.1007/s11357-025-01859-4

**Published:** 2025-09-05

**Authors:** Renee C. M. A. Raijmann, Huiberdina L. Koek, Marielle H. Emmelot-Vonk, Willem R. P. Agema, Angele P. M. Kerckhoffs, Carolina J. P. W. Keijsers

**Affiliations:** 1 Department of Geriatrics, Jeroen Bosch Ziekenhuis, ’s-Hertogenbosch, Kingdom of the Netherlands; 2 Department of Geriatrics, University Medical Center Utrecht, Utrecht, Kingdom of the Netherlands; 3 Department of Cardiology, Jeroen Bosch Ziekenhuis, ’s-Hertogenbosch, Kingdom of the Netherlands

**Keywords:** Comprehensive geriatric assessment, Geriatric consultation, Recommendation acceptance rates, Quality of care improvement, Cardiovascular disease

## Abstract

As patients with cardiovascular disease age, the need for cardiogeriatric care grows. Therefore, we aimed to identify facilitators and hindrances to the implementation of cardiogeriatric care by analysing geriatric recommendations content and acceptance rates. This retrospective cohort study included 100 patients aged > 85 years who received geriatric consultation and were admitted to a cardiology department. A random sample was generated, of which half received proactive geriatric consultation and half received usual care (geriatric consultation upon request). The recommendation content was categorized into geriatric domains (physical, mental, functional, social, and existential), and recommendation uptake was categorized into seven categories (e.g., accepted, declined by patient). Additionally, factors associated with acceptance rates were analysed using univariate logistic regression. The study population (mean age 88, 60% female) received 310 recommendations of which 56% were accepted. The acceptance rate was significantly higher if recommendations belonged to the functional (OR 3.10 (95% CI 1.41–6.82)), social (OR 5.55 (95% CI 2.40–13.00)), and existential domain (OR 6.09 (95% CI 2.62–14.16)) compared to the physical domain. Furthermore, higher acceptance rates were observed if recommendations were communicated verbally instead of electronically (OR 2.16 (95% CI 1.30–6.70)), when a clear tone was used instead of a doubtful one (OR 1.90 (95% CI 1.01–3.56)), and if the recommendation was concisely documented (OR 1.11 (95% CI 1.04–1.20) per average 10 words decrease). In this study, the acceptance rate of geriatric recommendations for older patients with cardiac disease was low. Recommendations regarding the functional, social, and existential domains had the highest acceptance rates. To improve acceptance rates, recommendations should be communicated verbally, documented concisely, and a doubtful tone should be avoided.

**Supplementary information:**

The online version contains supplementary material available at 10.1007/s11357-025-01859-4.

## Introduction

Due to advances in acute cardiac care, the peak prevalence of cardiovascular disease has shifted to the oldest old population (32.8% females, 39.6% males) [1, 2]. In this age group, it is common for patients with cardiac disease to experience geriatric syndromes such as functional dependency, cognitive impairment, and increased fall risk. These syndromes constitute to frailty, a clinical condition characterized by a decline in physiological reserve and increased vulnerability to stressors. Frail patients are at heightened risk for adverse health outcomes, including hospitalisation, functional decline, and mortality [3, 4].

A holistic and multidisciplinary approach to care is therefore essential to address the complex needs of frail older adults and to mitigate the risk of poor outcomes. However, most healthcare professionals are not adequately trained to recognize or manage frailty and its associated syndromes [5]. As a result, critical issues such as depression, inappropriate medication use, delirium, and cognitive dysfunction often remain underdiagnosed or untreated in older cardiac patients [5–7].

Recognition of frailty in cardiac care has gained increasing attention in recent years. Consequently, experts have advocated for closer collaboration between cardiologists and geriatricians in the form of cardiogeriatric care [8, 9]. Although there is no current general definition of what cardiogeriatric care entails, in this paper, we refer to collaborative practices such as clinical heart failure units where cardiologists and geriatricians jointly treat hospitalized patients, or standard pre-operative outpatient evaluations of cardiothoracic surgery candidates by both a cardiologist and a geriatrician [9].

Geriatricians use a comprehensive geriatric assessment (CGA) to evaluate patients across multiple domains––medical, mental, functional, and social to develop a personalized care plan [10]. In non-cardiac patient populations, CGA implementation has been associated with improved health outcomes, including reduced length of hospital stay, fewer complications, and less functional deterioration during hospitalization [10–13].

Despite these positive findings, evidence supporting the effectiveness of CGA in older cardiac patients remains limited and inconsistent. Some studies suggest that, even when CGA-generated recommendations are appropriate, implementation may be suboptimal [14]. This lack of follow-through may diminish the potential benefits of CGA. Reported acceptance rates for CGA recommendations vary greatly, ranging from 33 to 72% [15–19]. These ranging acceptance rates have been described in different settings, like hospitalized [15, 18] outpatients [16, 19] or primary care patients [17], and in different patient populations, like cancer patients [16], HIV patients [19], and hospitalized patients [15, 17, 18]. Yet, in our literature search, we found no studies evaluating recommendation acceptance in hospitalized patients with cardio(vascular) disease. Furthermore, we found that studies differed in how data on recommendation acceptance were obtained and rated. In some of the previous studies, results could be affected by recall bias, as acceptance rates were collected using self-reporting from patients or clinicians. Additionally, information on the content of the recommendations and reasons for the low recommendation acceptance rates is often not explored in previous studies.

To address this gap, the present study aims to analyse the nature of CGA-based recommendations, evaluate their implementation rate, and identify factors associated with recommendation acceptance in a cohort of oldest old patients (85 +) hospitalized due to cardiac disease.

## Methods

### Study design, setting, and sample

**Fig. 1:**
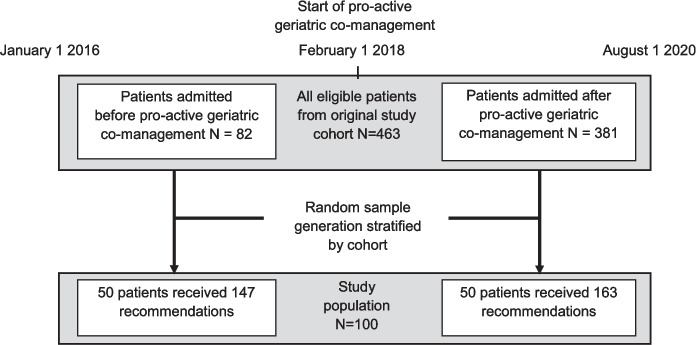
Flowchart of the selection of the study population

Figure 1 presents an overview of the study design. This retrospective cohort study aimed to describe the content and acceptance rate of geriatric recommendations provided to older patients admitted to the cardiology department. The study was conducted at the cardiac care unit and the cardiac nursing ward of a large teaching hospital in the Netherlands.

Patients aged 85 years and older were retrospectively included if they were admitted between 1 January 2016 and 1 August 2020 and had been evaluated by the hospital’s geriatric consultation team. There were no exclusion criteria.

This study builds upon a previously published investigation that compared proactive geriatric co-management with geriatric consultation on demand in a similar population [20]. As part of a quality improvement initiative, structured cardiogeriatric collaboration was introduced on February 1, 2018, for all cardiac inpatients aged 85 years and older.

A random sample of 100 patients was generated from the original study population. Due to the descriptive nature of the study, no sample size calculation was made. The researchers estimated that data saturation would be reached with the data of 100 patients. The sample generation was stratified by cohort (50 patients received usual care and 50 patients received proactive geriatric co-management). The stratification method was used to limit potential bias that the implementation of a proactive co-management intervention could have introduced regarding recommendation acceptance.

The regional Ethics Review Board (METC Brabant/20.435, #NW2020-78) deemed this study to fall outside the scope of the Dutch Law on Medical Research (WMO). As this was a retrospective study with no clinical impact on patients or their care, and none of the patients had a registered objection to the use of their data for scientific research, researchers did not contact patients or their family members for informed consent. Reporting was conducted in accordance with the Strengthening the Reporting of Observational Studies in Epidemiology (STROBE) guidelines.

### Description of care procedures

In the usual care cohort (before February 2018), patients received a geriatric consultation only upon request by the treating cardiologist. In contrast, after implementation of proactive co-management, all patients aged 85 years and above were routinely assessed by the geriatric team within 48 h of admission. In addition, a geriatric nurse made weekly rounds to provide support to cardiology ward nurses and address geriatric care concerns.

In both cohorts, the geriatric consultation followed the principles of a CGA, evaluating five core domains: medical, psychological, functional, social, and existential. Based on the findings of the CGA, the consultant team formulated tailored care recommendations, which were communicated directly to both the patient and their treating physician. Following the initial consultation, the geriatric consultant team could either conclude their involvement (single visit) or continue follow-up visits as clinically indicated (multiple visits).

Subcategories for each assessment domain and illustrative examples of recommendations are provided in Table 1.

**Table 1:** Description of geriatric domains, the recommendation subcategories, and examples of recommendations

Geriatric domain	Recommendation subcategory	Examples of potential recommendations
Physical	General medical advice	Management of electrolyte imbalances
	Medication review	Identifying inappropriate prescribing using STOP/START criteria
	Osteoporosis	Management of osteoporosis
Functional domain	Mobility and falls	Consult a physical therapist
	Rehabilitation	Indication for post-discharge rehabilitation
	Malnutrition	Consult a dietitian
	Dysphagia	Consult a speech-language pathologist
Mental domain	Delirium	(Non)pharmacological management of delirium
	Cognitive disorder	Analysis of cognitive disorders
	Mood disorders	(Non)pharmacological treatment of mood disorders
Social domain	Evaluate care needs	Evaluate with the caregiver what is necessary for discharge
	Consult social support	Consult social support to meet care needs
Existential domain	Advance care planning	Perform an advanced care planning conversation with patients with heart failure
	Start terminal care	Stop treatments that prolong life and focus on patient comfort

Throughout the entire study period, nurses on the cardiac ward systematically screened all patients for fall risk, delirium risk, and malnutrition using standardized national protocols [21]. When indicated, patients were referred to a physiotherapist or a dietician for additional in-hospital interventions.

### Variables and data collection

Baseline characteristics collected from the electronic medical records included the following: age, sex, residential situation, primary admission diagnosis, number of medications at admission, and frailty status as measured by the Clinical Frailty Scale (CSF, range − 9) [22], multimorbidity as measured by the Charlson Comorbidity Index (CCI) [23], history of cardiovascular diseases (such as ischaemic heart disease, heart failure, and chronic kidney disease) and history of dementia, fall history in the previous 6 months, risk of malnutrition assessed via the Short Nutritional Assessment Questionnaire (SNAQ) [24], and functional status based on the KATZ index of independence in activities of daily living (KATZ-6).

Geriatric recommendations were classified into five main domains––physical, functional, mental, social, and existential––and further categorized into predefined subcategories based on content, similar to previously published literature [25]. To minimize bias introduced by variation in wording among geriatric consultants, recommendations were counted by subcategory rather than by the total number of individual suggestions. For example, the recommendation ‘Implement non-pharmacological interventions from the delirium prevention protocol’ and a more detailed version ‘Improve orientation, encourage early mobilisation and enhance sleep hygiene’ were both categorized as a single recommendation under the subcategory ‘delirium’ within the mental domain.

Recommendation acceptance was retrospectively assessed by reviewing patient records for documented implementation within 6 months after discharge. One independent investigator (RR) analysed the full clinical record, including notes from physicians, nurses, social workers, and allied health professionals, as well as discharge letters, prescriptions, and diagnostic results. Acceptance was categorized into seven predefined groups: (1) accepted, (2) partly accepted, (3) forwarded to general practitioner, (4) declined by treating physician, (5) declined by patient, (6) not accepted due to clinical deterioration and start of terminal care, and (7) unclear data regarding acceptance. Operational definitions and examples of each category are detailed in Supplement 1.

To assess for any facilitating factors for recommendation acceptance, associations were explored between acceptance rate and patient factors and care-related characteristics. The patient factors were age, sex, frailty, multimorbidity, and polypharmacy. The studied care-related characteristics were the total number of recommendations, single or multiple visits, control vs intervention cohort (before and after proactive geriatric co-management), electronic or verbal communication of recommendations, and continuity of geriatric consultants. In addition to patient- and care-related variables, we explored the association between recommendation acceptance and several content-related characteristics of the recommendations themselves. Specifically, we examined (1) the average number of words per recommendation (per decline or increase of 10 words), (2) clarity of wording (classified as clear vs vague), and (2) alignment with the treating physician’s presumed clinical perspective (categorized as affirming vs contrasting). Definitions, classification criteria, and examples for each of these characteristics are provided in Supplement 2.

### Statistical analysis

Descriptive statistics were used to summarize baseline characteristics of the sampled study cohort and the full eligible population. Categorical variables were presented as frequencies and percentages, and continuous variables as medians with interquartile ranges (IQR). Differences between groups were assessed using the *χ*^2^ test for categorical variables and the Mann–Whitney *U* test for continuous variables, to evaluate whether the randomly selected sample was representative of the broader study population.

The acceptance rate was calculated as the proportion of fully accepted recommendations relative to the total number of recommendations, both overall and stratified by geriatric domain and subcategory. To assess the relationship between the number of recommendations per patient and the individual acceptance rate, a Spearman’s rank correlation was conducted.

Univariate logistic regression analyses were performed to examine the association between recommendation acceptance and the following independent variables: patient-related factors (age, sex, frailty status, multimorbidity, and polypharmacy) and care-related characteristics (total number of recommendations, single vs multiple geriatric consultant visits, control vs intervention cohort, electronic vs verbal communication). Acceptance was dichotomized into either accepted or not accepted (partly accepted, forwarded to general practitioner, not implemented due to start of terminal care, declined by patient, declined by physician, unclear acceptance). Odds ratios (ORs) with 95% confidence intervals (95% CI) were calculated. Statistical analyses were performed using IBM SPSS Statistics for Windows Version 29 [26]. A two-sided *p* value of 0.05 was considered statistically significant.

## Results

### Patient population

Table 2 describes the baseline characteristics of the sampled study population and the original patient population. The random selection procedure was successful as no significant differences were observed in baseline characteristics between the sample and the original populations. The study included 100 patients, 60% of whom were female. The mean age of the study population was 88 years. Chronic kidney disease was the most prevalent cardiovascular disease (47%), followed by arrhythmias (43%) and ischaemic heart disease (39%). For the prevalence of other cardiovascular diseases, see Table 2. Most patients were classified as frail according to the Clinical Frailty Scale (CFS; 63% with a score of 5 or higher). When comparing the patient characteristics between the control and intervention cohorts, we found that patients in the intervention cohort had a lower median CSF score (5.5 vs 6.0, *p* < 0.05) and were less likely to have fallen in the past 6 months (22% vs 42%, *p* < 0.05).

**Table 2:** Baseline characteristics of the study population

	Sampled study population (*N* = 100)	All eligible patients (*N* = 463)
Demographics		
Age median (IQR)	88 (14)	88 (4)
Female *N *(%)	60	282 (61)
Residential situation		
Community *N *(%)	78	387 (84)
Institutionalized *N *(%)	22	76 (16)
Comorbidities		
CCI median (IQR)	6.0 (2)	6 (2)
History of		
Heart failure *N *(%)	31	136 (29)
Ischaemic heart disease *N *(%)	33	137 (30)
Arrhythmia *N *(%)	46	201 (43)
Heart valve disease *N *(%)	18	107 (23)
Peripheral artery disease *N *(%)	18	54 (12)
Diabetes mellitus *N *(%)	19	112 (24)
Chronic kidney disease *N *(%)	46	216 (47)
Dementia *N *(%) (*N* = 99)^†^	27	70 (15)
Geriatric parameters		
Clinical frailty scale median(IQR)	6 (2)	6 (2)
Polypharmacy		
Minor (0–4) *N *(%)	24	120 (26)
Major (5–9) *N *(%)	48	233 (50)
Severe (≥ 10) *N *(%)	26	107 (23)
Fall history *N *(%) (*N* = 90)^a^	32	149 (32)
SNAQ score median(IQR) (*N* = 92)^a^	0 (1)	0 (1)
KATZ score median(IQR) (*N* = 89)^a^	2 (3.5)	1 (2)
History of delirium *N *(%) (*N* = 88)^a^	27	101 (22)

Description of baseline characteristics of the sampled study population and all eligible patients from the original cohort study

*IQR* interquartile range, *CCI* Charlson comorbidity index, *SNAQ* short nutritional assessment questionnaire

^a^Lower total number of patients due to missing data

### Content of recommendations

**Fig. 2:**
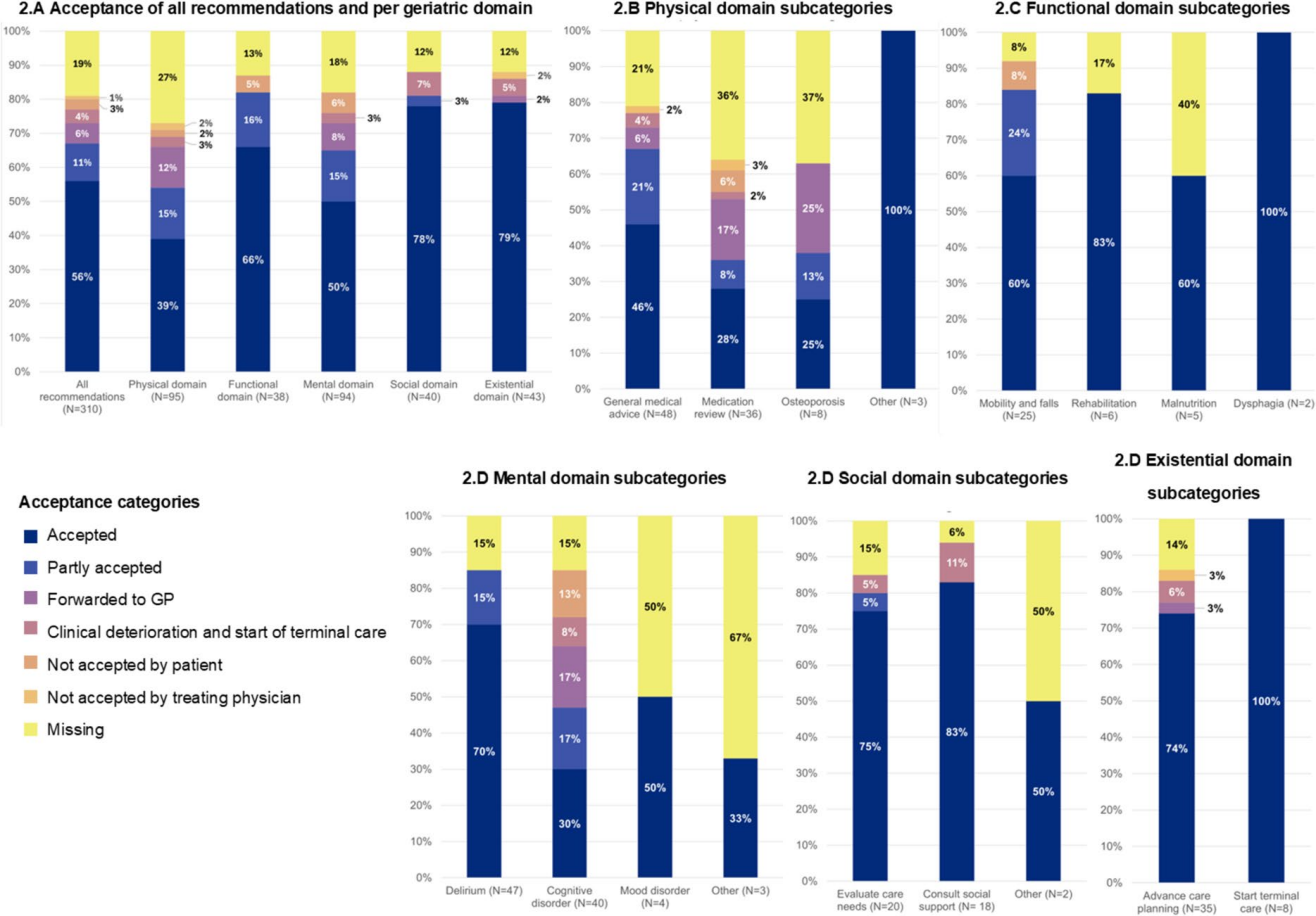
Distribution of acceptance rates across recommendations for different geriatric domains. Distribution of acceptance rates across **A** the total population and the geriatric domains, **B** subcategories of the physical domain, **C** subcategories of the functional domain, **D** subcategories of the mental domain, **E** subcategories of the social domain, **F** subcategories of the existential domain. Abbreviations; GP general practitioner

The geriatric team formulated a total of 310 recommendations for the 100 patients in the study population. The average number of recommendations per patient was 3.1 with a standard deviation (SD) of 1.4. The distribution of recommendations across the geriatric domains and subcategories is depicted in Fig. 2. Most recommendations focused on the physical and mental domains.

Within the subcategory medication review, the content of recommendations was further explored. The majority of medication review recommendations (63%) involved discontinuation of medications, followed by switching medications (18%). Starting medications (8%) or adjusting a dose (6%) were the least frequently recommended.

In the subcategory general medical advice, most recommendations addressed the analysis and treatment of infections (26%), followed by recommendations regarding anaemia (16%), electrolyte disturbances (11%), kidney injury (9%), dyspnoea management (9%), pain management (7%), and thyroid disorders (7%). The content of the other 15% was highly heterogeneous.

### Acceptance rate of recommendations

Figure 2A presents the acceptance rates of recommendations by domain for the entire study population. Overall, 56% of the recommendations were accepted, and 11% of the recommendations were partly accepted. Additionally, a proportion of recommendations were forwarded to the general practitioner (6%). In some cases, patients clinically deteriorated and received terminal care before recommendations could be implemented (4%). Explicit decline of recommendations was only 4% (1% by treating physician and 3% by patient). A considerable portion of recommendation acceptance was categorized as unclear (19%), which was most apparent in the physical domain (27%).

Figure 2B–D further illustrates how the acceptance rates are distributed across the recommendations for different geriatric domains and their subcategories. The recommendation subcategories with the highest acceptance rates were referral to rehabilitation facilities (83%), consult social support (83%), and start terminal care (100%). The lowest acceptance rates were found for the subcategories medication review (28%), osteoporosis (25%), and cognitive disorders (30%). These rates were low, because a considerable percentage of these recommendations was forwarded to the general practitioner (ca. 17%, 25%, 17%). Furthermore, the recommendations from the cognitive disorder subdomain were most often declined by patients themselves (13%). Other recommendations patients often refused were recommendations regarding mobility and falls (8%) and recommendations from the medication review (6%). Examples of recommendations patients often refused were the following: to stop benzodiazepines due to fall risk (subdomain mobility and falls) or discontinue proton pump inhibitors without a clear indication (medication review). One percent of recommendations was explicitly refused by the treating physicians, for which they explained their reasons.

### Factors associated with acceptance rate

Multiple patient and care-related factors were analysed on whether they were associated with recommendation acceptance. There was no significant association between the acceptance rate and the number of recommendations provided per patient (Spearman’s coefficient 0.096, *p* = 0.36). The associations for the other factors are presented in Table 3. There was a significant association between the geriatric domain of the recommendation and the acceptance rate. Recommendations for the functional, social, and existential domains were more likely to be accepted than recommendations for the physical domain (OR 3.1 (95% CI 1.41–6.82); OR 5.55 (95% CI 2.4–13); OR 6.09 (95% CI 2.62–14.16), respectively). There was no significant difference in acceptance rate between the physical and mental domains. Furthermore, recommendations that were communicated verbally were more likely to be accepted than recommendations communicated using only the electronic patient file (OR 2.16 (95% CI 1.3–6.7)). Using a neutral or directive tone was also associated with a better acceptance rate when compared to unclear or doubtful word use (OR 1.90 (95% CI 1.01–3.56)). Lastly, the acceptance rate increased by 11% for each reduction of 10 words used on average per recommendation (OR 1.11 (95% CI 1.04–1.20) per ± 10 words). This means that, as the number of words per recommendation decreased, the acceptance rate became higher. Patient characteristics (age, sex, frailty scores, multimorbidity, and polypharmacy) were not associated with acceptance rate. Similarly, there were no significant associations between acceptance rates and other care-related factors (single vs multiple visits, intervention vs control cohort (proactive co-management vs usual care), or continuity of care).

**Table 3:** Acceptance rate according to patient and care-related factors

	Odds ratio (95% CI)	***p*** value
Patient characteristics		
Age	1.01 (0.93–1.09)	0.90
Sex		
Male	1	-
Female	1.40 (0.85–2.12)	0.21
Clinical Frailty Scale	1.11 (0.92–1.33)	0.29
Charlson Comorbidity Index	1.00 (0.88–1.14)	0.96
Number of medications	0.96 (0.90–1.03)	0.24
Care characteristics		
Intervention type		
One-time visit	1	-
Multiple visits	1.03 (0.62–1.71)	0.90
Proactive geriatric co-management		
Control cohort	1	-
Intervention cohort	0.99 (0.63–1.55)	0.95
Communication of recommendation		
Via electronic patient file	1	-
Verbally via phone	2.16 (1.30–6.70)	< 0.01**
Continuity of care		
1 or 2 consultants	1	-
3 or more consultants	1.47 (0.84–2.56)	0.17
Average number of words per recommendation (± 10 words)	1.11 (1.04–1.20)	< 0.01**
Choice of words		
Unclear/doubtful word use	1	-
Neutral/directive word use	1.90 (1.01–3.56)	0.05*
Content of recommendation		
Affirmative	1	-
Contrasting	0.89 (0.57–1.40)	0.60
Domain of recommendation		
Physical domain	1	-
Functional domain	3.10(1.41–6.82)	< 0.01**
Mental domain	1.61 (0.90–2.88)	0.11
Social domain	5.55 (2.40–13.00)	< 0.01**
Existential domain	6.09 (2.62–14.16)	< 0.01**

Odds ratios were calculated using logistic regression to assess for any associations between recommendation acceptance and any patient or care-related characteristics

*95% CI* 95% confidence interval

^*^p < 0.05; **p < 0.01

## Discussion

This study showed a low acceptance rate of recommendations (56%) for patients aged 85 years and older admitted to a cardiology ward. Patients declined 3% of the recommendations, mostly in the subcategory of cognitive analysis. Only 1% of recommendations were explicitly refused by the treating physicians. Acceptance rates were associated with the content and communication of recommendations. Recommendations on the functional, social, and existential domains showed higher acceptance rates than recommendations regarding the mental and physical domains. Furthermore, recommendation acceptance was better for verbally communicated recommendations and recommendations that were documented concisely or used a directive/neutral tone.

### Recommendation acceptance rates

Acceptance rates from other studies varied from 33 to 72% [15–19]. These studies were performed in many different patient populations and care settings, for example, an inpatient geriatric consultation team on medical and surgical wards [15, 18], outpatient geriatric assessment in older patients with cancer [16] or HIV [19], or a geriatric assessment in older bedridden patients at home with recommendations provided to the general practitioner [17].

Studies with the most similar design, an inpatient geriatric consultation team, had better overall acceptance rates of 70% [18] and 72% [15]. Morin et al. [15] probably had the highest recommendation acceptance, because they combined the acceptance categories of partly accepted and totally accepted recommendations, thus possibly overestimating the number of recommendations that were actually completely implemented. Another factor was that they called patients after 3 months of follow-up to assess recommendation adherence which enabled them to evaluate whether recommendations forwarded to the GP were implemented or not. Though a patient recall can be subject to recall bias which limits the reliability of this data. In Deschodt et al., researchers recruited geriatric consultant teams from different hospitals before study initiation. The researchers acknowledge that it is likely that teams with a perceived higher adherence rate were more likely to participate in the study, which might limit the generalizability of the results. Additionally, the recommendation acceptance was obtained through self-reporting, which is also subject to recall bias. These factors might have resulted in better (documentation of) recommendation acceptance, leading also to a lower proportion of unclear acceptance and higher acceptance rates [18].

Another potential contributing factor to our lower acceptance rate is that the geriatric consultant teams’ composition was different between studies. In our study, the team consisted of junior doctors and residents who were aided by geriatric nurses and supervised by an experienced geriatrician. Whereas the geriatric consultant team of the study by Deschodt et al. consisted of nurses and paramedics with an average of 11 years of experience working in the team [18]. This hypothesis is supported by the finding by Deschodt et al. that the consultants’ experience years and acceptance rate were positively associated (OR 1.34 (95% CI 1.04–1.72)) [20]. Unfortunately, we did not have the data to perform these additional analyses.

### Factors associated with recommendation acceptance

We identified several factors that were associated with the acceptance rate of the recommendations. The type of geriatric domain of the recommendations was significantly associated with the acceptance rate. Recommendations concerning the functional, social, and existential domains were more likely to be accepted than recommendations of the physical and mental domains. This was mostly caused by low acceptance for recommendations concerning medication reviews, osteoporosis, and cognitive impairment which was also found in other research [15, 16, 18].

A considerable portion of the recommendation acceptance for these subdomains was categorized as ‘forwarded to a general practitioner’. It is not clear from the hospital’s electronic patient records whether these recommendations were implemented by the general practitioners or not. This illustrates a barrier to CGA implementation that has been discussed in previous literature: the lack of communication between primary and secondary care providers. Insufficient communication between staff carrying out the geriatric assessment and general practitioners can create cynicism about the relevance of the assessment efforts to care [27].

Another portion of the recommendation acceptance for the subdomains medication review and cognitive impairment was categorized as ‘declined by patients’. Previous research has demonstrated that adherence to CGA recommendations can be hindered by patients’ willingness to adhere to these recommendations [27, 28]. Patients may not view certain issues as health problems, or they may not perceive the beneficial value of geriatric services. Additionally, sometimes wrong perceptions or fear of stigma can prevent patients from visiting geriatric care services such as a ‘memory clinic’ [27, 28].

The style of communication of the recommendations was also associated with the acceptance rate. Recommendations that were communicated verbally instead of electronically were overall better accepted. Deschodt et al. also analysed this factor but did not find an association (OR 0.96 (95% CI 0.41–2.27)) [18]. Furthermore, concisely formulated recommendations (the acceptance rate increases 11% per 10-word decrease of average words used per recommendation) were more likely to be accepted than long-winded ones, and recommendations using a neutral or directive tone had a higher acceptance rate than recommendations with an unclear or doubtful word use. These factors have not yet been evaluated in other research.

Previous studies found that the average number of recommendations was associated with acceptance rate. Two studies found that, as the average number of recommendations per patient increased, the acceptance rate decreased [15, 18]. This finding was not confirmed in our study, which might be caused by the fact that the average number of recommendations per patient in our study was already quite low.

Furthermore, another study found that, if the same geriatrician who performed the CGA was involved with its implementation, the acceptance rates also improved (OR 4.8 (95% CI 2.3–9.6)) [17]. Similarly, this result was not reproduced in our study. However, in the study by Greenbom et al. [17], the treating physician always remained the same general practitioner. Whereas in our study, even though the geriatric consultant remained the same, the treating cardiac physician often changed, for example, when patients were transferred from the cardiac care unit to the general cardiac ward. So, the continuity of care did not depend only on the continuity of geriatric care consultants.

Bitas et al. performed a qualitative study using a survey conducted amongst physicians treating older patients with HIV [19]. The survey evaluated the physician’s opinions and experiences regarding geriatric consultation and recommendations. From the survey results, they found that the majority of physicians regarded the geriatric consult as very useful and usually or always implemented the recommendations they received. However, the recommendation acceptance rate in this study was actually low (32%). So, there was a discrepancy between the acceptance rate assessed by the researchers and the acceptance rate perceived by the treating physicians. Furthermore, the most important reason they listed for not following a recommendation was that they thought the recommendations were not feasible. None of the physicians who filled out the survey said that there were too many recommendations, that the recommendations were not clear, or that they did not agree with the content of the recommendations. Similarly, in our study, the percentage of recommendations that were explicitly refused by treating physicians was also very low (1%).

### Strengths and limitations

This was an observational study that evaluated recommendations and their acceptance rate based on a retrospective chart review. The categories for recommendation content and acceptance were carefully chosen to best describe and understand the process of recommendation acceptance. This allowed us to evaluate how communicative factors influence recommendation acceptance in more detail compared to previous research. Additionally, we identified no other similar studies performed specifically in an oldest old (85 +) cardiovascular patient population, which is a very relevant and fast-growing patient population in common practice that is often not included in research.

However, the retrospective study design introduces a risk of bias. Data was retrospectively collected by one researcher from electronic records which could have been misinterpreted. To counteract this, we have attempted to make very clear definitions of all classifications and categories of variables as provided in the supplementary documents. Additionally, by including patients from a single centre, the generalizability of this study is limited. Furthermore, there was a significant portion of recommendations where the acceptance was unclear. To address this, we evaluated recommendation acceptance using an elaborate evaluation of the patient charts, including physician, nurse, paramedic, and social workers’ notes, as well as imaging and laboratory findings. As we found no chart evidence for the implementation of these recommendations, it is highly likely that these recommendations were not implemented. Furthermore, there was a significant portion of recommendations where the acceptance was unclear. To address this, we evaluated recommendation acceptance using an elaborate evaluation of the patient charts including physician, nurse, paramedic, and social workers’ notes, as well as imaging and laboratory findings. As we found no chart evidence for the implementation of these recommendations, it is highly likely that these recommendations were not implemented. As the outcome of unclear acceptance was included in the ‘not accepted’ outcome in our analysis, this missing data might have led to an overestimation of the non-accepted rates.

### Recommendations

This current retrospective study provides more insights into which practical recommendations could be given to improve acceptance rate. It would be highly interesting to study this further in an intervention study that investigates whether systematic implementation of these practical implications in clinical practice results in higher acceptance rates.

Furthermore, in order to better understand the barriers to CGA implementation, it is recommended that future studies on this topic be performed prospectively with follow-up in the primary care setting, rather than retrospectively. Additionally, qualitative, prospective analysis on both barriers and facilitators for the acceptance rate would be of great value to confirm our results and further expand our knowledge to better understand why recommendations are not accepted. Potential factors that have not been studied in previous research could be included such as hospitalization length, a patient’s education level, and the acceptance culture in the hospital.

Our study can trigger clinicians to be more aware of not only what they recommend but also how they communicate their recommendations. The first step to change is the realization that something actually can be improved. A simple change might be to take up the habit of considering how you can document your recommendations in a shorter or clearer way, or of calling the treating physician after a consultation to inform them of your findings and recommendations. Two practical examples of phrasing the same recommendation are as follows:

1. Not clear nor concise:
  Please consider performing additional lab (infection lab and urine analysis) and/or imaging studies (chest X-ray) to find the focus of the delirium.
  You could also try to improve the patient’s orientation by making sure he is using his glasses, hearing aids, and using a calendar which indicates the current date and day of the week.
  Additionally, you might monitor his delirium by performing a DOS score on a regular basis.
2. Clear and concise:
  Find the focus of the delirium (infection lab, physical examination, urine analysis, and chest X-ray).
  Implement non-medication interventions from the local delirium prevention protocol.
  Perform a DOS score each shift.

## Conclusions

This retrospective descriptive study found that the acceptance rate of geriatric recommendations was low (56%) in the oldest old patients (85 +) admitted for a cardiac disease. Several factors regarding the content and communication of the recommendations were associated with a higher acceptance rate. Recommendations regarding the functional, social, and existential domains had higher acceptance rates than recommendations from the physical and mental domains. Furthermore, recommendations were more likely to be accepted when they were documented concisely, with a neutral and direct tone, and verbally discussed with the treating physician. Though these results should be interpreted carefully considering the study design and patient selection, these recommendations can potentially aid clinicians in improving recommendation acceptance rates in their daily practice.

## Supplementary information

Below is the link to the electronic supplementary material.

Supplementary file (DOCX 16.6 KB)

## Data Availability

The data analysed in this study is not publicly available as the participants of this study did not give written consent for their data to be shared publicly.
