# Supplementary material for: Short communication and clear content of geriatric recommendations matter! Evaluation of geriatric recommendation acceptance in patients aged ≥ 85 years hospitalized for cardiac conditions
Source: GeroScience. 2025 Sep 5;48(3):3715–27. doi: 10.1007/s11357-025-01859-4 (PMC13356211; doi:10.1007/s11357-025-01859-4)
Supplement: Supplementary file 1 — Supplementary file (DOCX 16.6 KB) [file 11357_2025_1859_MOESM1_ESM.docx]

Supplement 1: Description of acceptance categories.

| **Acceptance category** | **Description and examples** |
| --- | --- |
| Accepted | Recommendation is accepted by both the treating physician and the patient and implemented during the course of the admission. |
| Partly accepted | If multiple individual recommendations all fall under 1 subcategory, they are counted as 1 recommendation. If only a part of these individual recommendations was accepted and implemented they are categorized as partly accepted. For example: if the recommendation was ‘search for the origin of the infection using a chest x-ray, blood and urine culture’ and only a chest x-ray was performed, but not the cultures, the recommendation in subcategory general medical advice was categorized as partly accepted. |
| Forwarded to general practitioner | If the treating cardiac physician or the geriatric consultant forwarded the recommendation to the general practitioner either through discharge letter or by phone, the recommendation was categorized as forwarded to the general practitioner. As there was no data on the uptake of these recommendations, it is unclear whether these recommendations were implemented. |
| Not accepted by patient | Recommendations are discussed with the patient by the geriatric consultant team. If the patient waives the recommendation it was categorized as not accepted by patient. |
| Not accepted by treating physician | Recommendations were categorized as not accepted by treating physician if the treating physician documented that they did not follow the geriatric recommendation. Often the reason for deviating from the recommendation was further corroborated. |
| Not accepted due to reduced life expectancy | Most recommendations are formulated in the beginning of a hospital admission. However, if a patient unexpectantly clinically deteriorates during the hospital admission, initial recommendations might become irrelevant. For example, the recommendation ‘Outpatient analysis of potential cognitive impairment’ becomes irrelevant if a patients dies due to a nosocomial infection on top of their decompensated heart failure. |
| Missing | In case we could not find any sign in the electronic patient file the acceptance or implementation of the recommendation it was classified as missing. It is assumable these recommendations did not impact clinical care. |

Supplement 2: Description of care and recommendation characteristics and explanation of chosen cut off values if applicable.

| **Care characteristics** | **Description and explanation of cut off values** |
| --- | --- |
| Number of recommendations | The total number of recommendations given during the patients admission. The number of recommendations was cut off at 3, which was the median number of recommendations for all included patients. |
| Consultation versus co-management | Consultation: the geriatric consultant team only visited the patient once and gave recommendations without follow up.  Co-management: the geriatric consultant team visited the patient multiple times during the hospital admission. |
| Control vs intervention cohort | Control cohort: patients admitted to the cardiac department between 1 January 2016 and 31 January 2018, before the start of the standard cardio-geriatric collaboration.  Intervention cohort: patients admitted to the cardiac department between 1 February 2018 and 1 August 2020, after the start of the standard cardio-geriatric collaboration. |
| Electronical or verbal communication | The consultant team uses a standard text in which they have to fill out how the recommendations are communicated; via electronic patient file (electronical) or by phone (verbal). If this part of the standard text was not filled out; this variable was scored as missing. |
| Continuity of consultants | The number of different consultants were counted throughout the entire admission. This variable was only applicable to patients receiving co-management (where there was a chance for multiple consultants). As the geriatric consultant team consists of two people (a physician and a specialised nurse) the cut off for this variable was put at 2. |
| Mean number of words per new recommendation | The mean number of words per new recommendation was calculated by adding the number of words of the conclusion to the total number of words of all recommendations given during the admission and dividing them by the number of recommendations given during the admission. This was chosen because the conclusion is often quite long and is repeated in each consult which adds to wordiness of the consult. The mean number of words were analysed in groups of 10 words to better estimate the scale of the effect size. |
| Wording of recommendation | The wording was categorized as either neutral/clear or doubtful/unclear.  The recommendations were rated as doubtful/unclear in case only a remark was made without an actual recommendation, or if it contained words like ‘consider’, ‘evaluate’, ‘potentially’ and ‘maybe’ or if a question mark was used. All other recommendations were rated as neutral/clear. |
| Contrasting or new content of recommendation | The content of the recommendation was rated as new or contrasting in case the topic of the recommendation was not previously documented as an issue by the treating cardiac physician or if the cardiac physician documented a contrasting view to the recommendation. |
